# Uniportal-VATS vs. open McKeown esophagectomy: Surgical and long-term oncological outcomes

**DOI:** 10.3389/fsurg.2023.1103101

**Published:** 2023-02-27

**Authors:** Dania Nachira, Maria Teresa Congedo, Giuseppe Calabrese, Diomira Tabacco, Leonardo Petracca Ciavarella, Elisa Meacci, Maria Letizia Vita, Giovanni Punzo, Filippo Lococo, Federico Raveglia, Marco Chiappetta, Venanzio Porziella, Angelo Guttadauro, Ugo Cioffi, Stefano Margaritora

**Affiliations:** ^1^ Department of General Thoracic Surgery, Fondazione Policlinico Universitario “A. Gemelli”, IRCCS, Università Cattolica del Sacro Cuore, Rome, Italy; ^2^ Department of Anesthesiology and Intensive Care Medicine, Fondazione Policlinico Universitario “A. Gemelli”, IRCCS, Università Cattolica del Sacro Cuore, Rome, Italy; ^3^ Department of Thoracic Surgery, ASST Monza, San Gerardo Hospital, Monza, Italy; ^4^ Department of Medicine and Surgery- School of Medicine and Surgery, Università Degli Studi di Milano Bicocca, Milan, Italy; ^5^ Doctorate Professor, University of Milan, Milan, Italy

**Keywords:** uniportal-VATS, mckeown esophagectomy, esophageal cancer, disease-Free survival, oncological outcomes, CUSUM, learning curve

## Abstract

**Background:**

Till now there are very few reports about surgical results of Uniportal-VATS esophagectomy and no one about long-term outcomes. This study is the first comparing surgical and oncological outcomes of Uniportal-VATS with open McKeown esophagectomy, with the largest reported series and longest oncological follow-up.

**Methods:**

The prospectively collected clinical, surgical and oncological data of 75 patients, undergone McKeown esophagectomy at our Thoracic Surgery Department, from January 2012 to August 2022, were retrospectively analyzed. Nineteen patients underwent esophagectomy by thoracotomy and reconstruction according to McKeown technique while 56 by Uniportal-VATS approach. Gastric tubulization was performed totally laparoscopic or through a mini-laparatomic access and cervical anastomosis was made according to Orringer's technique.

**Results:**

The mean operative thoracic time was similar in both accesses (102.34 ± 15.21 min in Uniportal-VATS vs. 115.56 ± 23.12 min in open, *p*: 0.646), with a comparable number of mediastinal nodes retrieved (Uniportal-VATS:13.40 ± 8.12 vs. open:15.00 ± 6.86, *p*: 0.275). No case needed conversion from VATS to open. The learning curve in Uniportal-VATS was completed after 34 cases, while the Mastery was reached after 40. Both approaches were comparable in terms of minor post-operative complications (like pneumonia, lung atelectasis, anemization, atrial fibrillation, anastomotic-leak, left vocal cord palsy, chylothorax), while the number of re-operation for major complications (bleeding or mediastinitis) was higher in open group (21.0% vs. 3.6%, *p*: 0.04). Both techniques were also effective in terms of surgical radicality and local recurrence but VATS approach allowed a significantly lower chest tube length (11.89 ± 9.55 vs. 25.82 ± 24.37 days, *p*: 0.003) and post-operative stay (15.63 ± 11.69 vs. 25.53 ± 23.33, *p*: 0.018). The 30-day mortality for complications related to surgery was higher in open group (*p*: 0.002). The 2-, 5- and 8-year survival of the whole series was 72%, 50% and 33%, respectively. Combined 2- and 5-year OS in Uniportal-VATS group was 76% and 47% vs. 62% and 62% in open group, respectively (Log-rank, *p*: 0.286; Breslow-Wilcoxon: *p*: 0.036). No difference in DFS was recorded between the two approaches (5 year-DFS in Uniportal-VATS: 86% vs. 72%, *p*: 0.298). At multivariate analysis, only pathological stage independently affected OS (*p*: 0.02), not the surgical approach (*p*: 0.276).

**Conclusions:**

Uniportal-VATS seems to be a safe, feasible and effective technique for performing McKeown esophagectomy, with equivalent surgical and long-term oncological results to standard thoracotomy, but with a faster and unharmed recovery, and a quite short learning curve.

## 1. Introduction

Esophagectomy still represents the crucial therapeutic choice of resectable esophageal cancers in multimodal treatments. Open esophagectomy, being a high invasive surgery with 2 or 3 access fields involved, it is burdened by a high post-operative mortality, with about 50% of patients at risk for developing post-operative respiratory complications and long hospital stay (1).

In the last 20 years, minimally invasive esophagectomy (MIE) has been proven to be superior to open esophagectomy (OE) in surgical and short-term results, reducing morbidity, however the oncological outcomes are still controversial and required further verification by randomized trials (2).

In this scenario and in the field of MIE, the role of Uniportal-Video assisted Thoracic Surgery (VATS) esophagectomy is even more debated and right now very limited reports (mainly cases series with short-term results or surgical technique papers) are available to can address this point (3, 4).

The main reason of lack of study on long-term outcomes in Uniportal-VATS esophagectomy are ascribable to the fact that it is considered a surgical demanding technique, with a quite longer learning curve, that requires not only a large experience in esophageal surgery and posterior mediastinum manipulation but also good surgical skills in single-access approach and dexterity in hand-eye coordination (5, 6).

Based on our long experience in esophageal surgery and Uniportal-VATS field, in this paper we reported the surgical and long-term oncological outcomes of Uniportal-VATS approach compared with thoracotomy for performing McKeown esophagectomy.

## 2. Materials and methods

The prospectively collected clinical, surgical and oncological data of consecutive 75 patients, undergone McKeown esophagectomy at our Thoracic Surgery Department, from January 2012 to August 2022, were retrospectively analyzed. All patients had a diagnosis of upper, middle or lower esophageal cancer.

Among these, 19 underwent esophagectomy by thoracotomy (the performed approach at our center from January 2012 to November 2016) and reconstruction according to McKeown technique while 56 patients underwent Uniportal-VATS approach (December 2016 – August 2022), that has become the preferred approach at our center for major and minor thoracic procedures, since June 2016. All patients undergone other esophageal reconstructions (as Ivor-Lewis esophagectomy open or VATS) along the study period were excluded to reduce selection biases related to different surgical procedures.

The diagnostic and preoperative evaluations included: esophagogastroduodenoscopy (EGD) for diagnosis and endoscopic ultrasound (EUS) to evaluate T-stage and nodal involvement, Total-body computed tomography (CT) and PET-CT for disease stage, pulmonary function test, cardiac tests and blood analyses.

While indication to neoadjuvant and/or surgical treatment of esophageal cancer may vary according to TNM stage and local institutions, at our center each case was discussed in a dedicated tumor board (involving oncologists, radiotherapists, thoracic and general surgeons) and, in agreement with recent guide-lines (7), patients with a IIB–IIIB stage (8th Edition of American Joint Committee on Cancer (AJCC) TNM staging system (8) underwent preoperative inductive radio/chemotherapy.

Post-induction re-evaluation and staging was done by PET-CT and EUS when necessary.

All patients signed an informed consent before surgery for the treatment of their clinical data.

### 2.1. Surgical technique

According to McKeown technique, radical esophagectomy and reconstruction include 3 surgical times: thoracic, abdominal and cervical one.

The main steps of each time (Uniportal-VATS thoracic approach, abdominal and cervical approaches) were already described in a previous paper (9) on the technique by our group.

According to our experience, a particular importance must be given to the position of patient on the operative table and to the use of operative table itself, during Uniportal-VATS.

Indeed, the patient lies on his left side, with the bed flexed down of 30–45° at the level of his V intercostal space. After blocking and ensuring the patient on the bed by a vacuum matrass, the bed is tilted about 45° toward patient's ventral side (where surgeons stand during the operation) and 30° in anti-Trendelenburg's position. These precautions, together with the location of the 4 cm Uniportal-VATS incision (on V intercostal space but more posterior than for lung surgery, on the anterior margin of latissimus dorsi, that is spared), give the possibility to have more space for the simultaneous use of several instruments through a small incision, and to better expose and dissect the posterior mediastinum, the esophagus itself and all mediastinal nodal stations.

On the contrary, in open thoracic approach, a lateral muscle sparing thoracotomy is performed at V intercostal spaces. The steps of esophageal dissection and mobilization are the same as in VATS surgery. In both groups, the thoracic duct was not always closed or clipped routinely during the study time.

A careful lymphadenectomy was performed in both approaches, removing all fatty tissue and nodes along esophagus, aorta, thoracic duct, pulmonary ligament, sub-carinal and upper para-tracheal space and Barety's space. In open approach, elettrocautery and clips were used to coagulate and seal lymphatic vessel, in VATS surgery the same energy device used for esophageal dissection was employed.

At the end of open esophagectomy, 2 chest tubes (28 Fr) were left in place through the VII (anterior apical drain) and VIII (posterior basal drain) intercostal spaces, instead of one (through the same incision) as in Uniportal-VATS approach.

The abdominal time was carried out open or laparoscopic, according to the period when the operation was performed at our center.

In each patient, a jejunostomy tube was placed at the end of surgery for early enteral nutrition.

### 2.2. Intra- and peri-operative management

Surgery was carried out in general anesthesia, with single-lung ventilation. For analgesic purpose, all patients underwent intercostal nerve blockade (in the incision space, one space above and 2–3 spaces below) by 5% ropivacaine (3–5 cc per space) under direct view by surgeon, at the end of thoracic time. An elastomeric pump was also used for intravenous administration of Tramadol (12.5 mg/h in VATS group) and Morphine (1 mg/h in open group) for 24 h. Patients were extubated immediately after surgery or the day after, in the intensive care unit, according to anesthesiological decision, based on patient's clinical condition and length of surgery.

All patients received post-operative intravenous antibiotics (second-generation cephalosporin, metronidazole and fluconazole).

Since the first post-operative day, the early mobilization of the patient was stimulated to enhance the recovery. Meanwhile, a progressive implementation of enteral nutrition was achieved by jejunostomy to obtain the correct metabolic intake according to the dedicated team of nutritionists.

An x-ray esophagogram was performed on V-VI post-operative day for evaluating transit of swallow and excluding cervical anastomotic leak, before restarting oral intake. The cervical drainage and last chest tube were removed after starting re-alimentation per os in absence of clinic-radiological complications.

### 2.3. Oncological follow-up

Patients were followed-up by a dedicated team of oncologists every 3 months for the first 2 years, then every 6 months in the following 3 years, and then annually from the 5th year. The radiological examinations used were neck and chest CT scan and complete abdomen ultrasound. Other specific blood markers or endoscopic evaluations were required by oncologist according to the case.

### 2.4. Statistical analysis

Continuous variables were expressed as mean and standard deviation, while categorical variables as absolute numbers and percentages (%). Kolmogorov–Smirnov test was used to evaluate normal distribution of data. Continuous variables were compared by independent sample Student's *t*-test if normal distributed or by Mann–Whitney *U*-test if not normal. Categorical variables were compared by Chi-squared test.

Overall survival (OS) was defined as time elapsed from surgery to death; disease-free survival (DFS) as time between surgery and first recurrence of disease in any site.

Survival and disease-free analyses were performed by Kaplan-Meier method; differences in survivals were evaluated by Log-Rank test or Breslow-Wilcoxon where indicated. Univariate analysis with a Cox proportional hazard model was conducted to evaluate prognostic factors. All covariates with *p* < 0.15 at univariate analysis were selected for Multivariate Cox regression analysis to assess factors independently affecting survival.

The CUSUM technique of the operative time was used to define the completion of our learning curve (CLC) in Uniportal-VATS esophagectomy.

The CUSUM series was defined as follows: ∑(Xi−X0), where Xi was an individual measurement [operative time of each case (ni)] and ×0 was a predetermined reference level, here set as the mean operative time of all cases. The CUSUM series was plotted against the consecutive procedures to calculate the point of downward inflection on the graph or cut-off value [the number of surgical procedures (ni) to overcome the LC, at which the highest value of ∑(Xi−X0) was reached].

Furthermore, a two-sided Bernoulli CUSUM chart was plotted to define the point of “mastery” of Uniportal-VATS esophagectomy, defined as the point where the operative time became consistent with the mean, without further significant changes in terms of mean operative time.

A *p*-value < 0.05 was considered statistically significant.

Statistical analysis was performed using IBM SPSS Statistics for Macintosh (version 25.0, IBM Corp, Armonk, NY, United States).

## 3. Results

The 56 patients operated on by Uniportal-VATS approach and the 19 patients by open technique were completely comparable in terms of main clinic-pathological characteristics, Table 1. In particular, no statistical difference was found in age, comorbidities, cancer histology, stage and neo-adjuvant and adjuvant therapies. The mean age in Uniportal-VATS group was 63.38 ± 10.17 years, while in open group was 63.95 ± 12.15 (*p*: 0.841). The main histology was adenocarcinoma in both groups (40 (71.4%) in Uniportal-VATS vs. 11 (57.9%) in open, *p*: 0.274). Thirty-three (58.9%) patients underwent neoadjuvant therapy in Uniportal-Vats group vs. 6 (31.6%) in open, *p*: 0.079; in particular, radiotherapy was the concurrent treatment in 57.1% of patients operated by Uniportal-VATS approach vs. 26.3% of open surgery, *p*: 0.539.

**Table 1:** Comparison of clinico-pathological characteristics of patients in 2 groups.

Variables	Uniportal-VATS Esophagectomy (#56 pts)	Open Esophagectomy (#19 pts)	*p*
Gender (male)	44 (78.6%)	12 (63.2%)	0.182
Age (years)	63.38 ± 10.17	63.95 ± 12.15	0.841
Smoking habitus	10 (17.9%)	5 (26.3%)	0.426
COPD	12 (21.4%)	4 (21.1%)	0.972
Diabetes mellitus II	6 (10.7%)	5 (26.3%)	0.097
Hypertension	13 (23.2%)	5 (26.3%)	0.784
Cardiovascular diseases	12 (21.4%)	5 (26.3%)	0.660
Histology			
Adenocarcinoma	40 (71.4%)	11 (57.9%)	0.274
Squamous cell	16 (28.6%)	8 (42.1%)	
Tumor extension (cm)	3.50 ± 2.42	4.02 ± 2.00	0.645
Tumor location			
Upper esophagus	4 (7.1%)	2 (10.5%)	0.593
Middle esophagus	31 (55.4%)	13 (68.4%)	
Distal esophagus	21 (37.5%)	4 (21.1%)	
Pathological stage:			
Complete response	2 (3.6%)	1 (5.3%)	0.734
I	20 (35.7%)	4 (21.1%)	
II	14 (25.0%)	6 (31.6%)	
III	15 (26.8%)	7 (36.7%)	
IVA (N2)	5 (8.9%)	1 (5.3%)	
Neoadjuvant therapy	33 (58.9%)	6 (31.6%)	0.079
Neoadjuvant chemotherapy	26 (46.4%)	4 (21.1%)	0.476
Neoadjuvant radiotherapy	32 (57.1%)	5 (26.3%)	0.539
Adjuvant therapy	20 (35.7%)	7 (36.8%)	0.808

**Figure 1:**
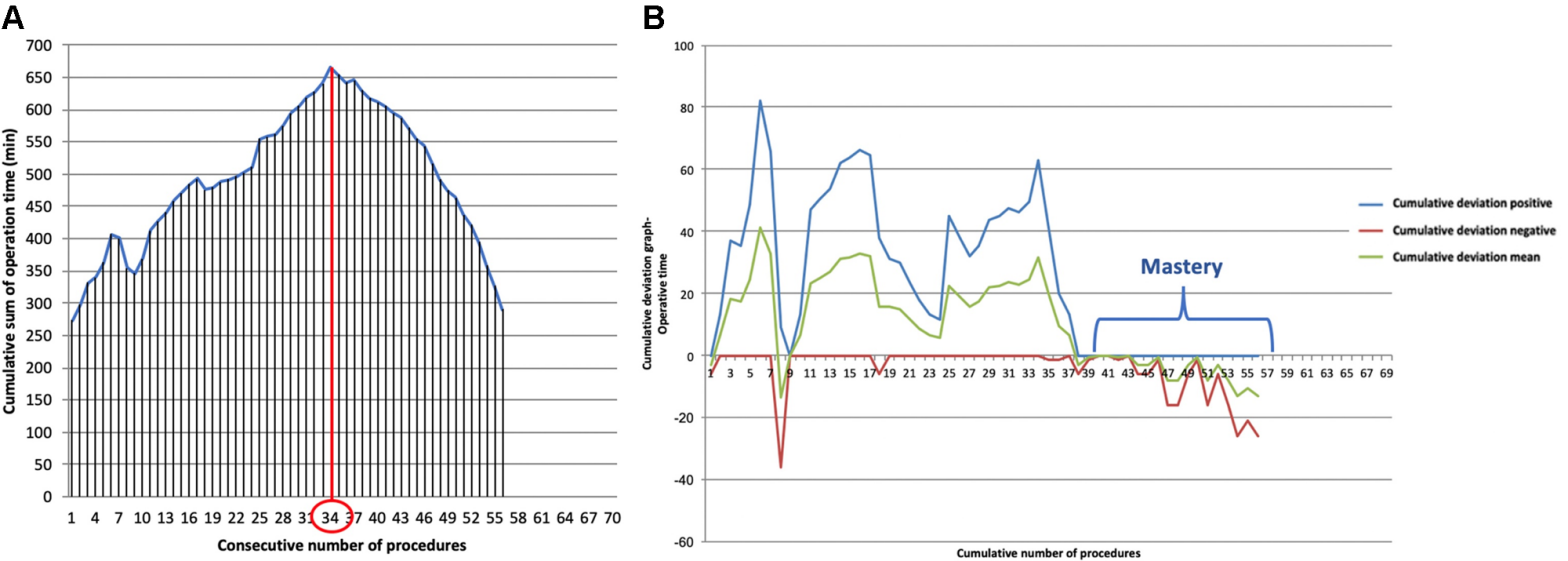
(**A)**. Cumulative sum (CUSUM) plot for the overall surgical time; the red circle is the CLC cut-off value on the plot of CUSUM analysis. CLC, completion of learning curve. (**B)**. Bernoulli cumulative deviation curves for CUSUM.

No case needed conversion in Uniportal-VATS group. The mean operative thoracic time was similar in both accesses (102.34 ± 15.21 min in Uniportal-VATS vs. 115.56 ± 23.12 min in open, *p*: 0.646), Table 2. In our experience, the learning curve of Uniportal-VATS esophagectomy was completed after 34 cases (CLC point in Figure 1A), while the mastery was reached after 40 cases (Figure 1B). All Uniportal-VATS esophagectomies were performed by the same operators (S.M., D.N.) during the study time.

**Table 2:** Comparison of surgical and short-term outcomes.

Variables	Uniportal-VATS Esophagectomy (#56 pts)	Open Esophagectomy (#19 pts)	*p*
Thoracic time (min)	102.34 ± 15.21	115.56 ± 23.12	0.646
Conversion	0	/	/
Number of thoracic nodes retrieved	13.40 ± 8.12	15.00 ± 6.86	0.275
Re-operation	2 (3.6%)	4 (21.0%)	**0**.**04**
*Post-operative minor complications:*			
Lung Atelectasis	3 (5.3%)	3 (5.4%)	0.148
Atrial fibrillation	7 (13.0%)	3 (5.4%)	0.716
Anemization	4 (7.1%)	1 (5.3%)	0.482
Pneumonia	7 (13.0%)	4 (21.1%)	0.363
Anastomotic leak	4 (7.1%)	4 (21.1%)	0.095
Chylothorax	3 (5.5%)	2 (10.5%)	0.435
Left vocal cord palsy	3 (5.3%)	1 (5.2%)	0.987
Myocardial infarction	0	1 (5.2%)	0.084
Chest drain (or last drain) removal (days)	11.89 ± 9.55	25.82 ± 24.37	**0**.**003**
Post-operative stay (days)	15.63 ± 11.69	25.53 ± 23.33	**0**.**018**
R + status	1 (1.8%)	0	0.655
Local recurrence	5 (8.9%)	2 (10.5%)	0.854
Thirty-day mortality	0	3 (15.7%)	**0**.**002**
Death of disease	15 (26.8%)	2 (10.5%)	0.135

A comparable number of mediastinal nodes was retrieved in Uniportal-VATS (13.40 ± 8.12) and open group (15.00 ± 6.86), *p*: 0.275, Table 2. Both approaches were also comparable in terms of minor post-operative complications (like pneumonia, lung atelectasis, anemization, atrial fibrillation, anastomotic-leak, left vocal cord palsy, chylothorax, Table 2), while the number of re-operation for major complications (bleeding or mediastinitis) or chylothorax was higher in open group (21.0% vs. 3.6%, *p*: 0.04). The 4 re-operations (21%) in open group were due to persistent chylothorax (in 2 cases) that required the surgical closure of thoracic duct, bleeding (in 1 case) or mediastinitis consequent to anastomotic leak (1 case), that needed a surgical toilette.

Both techniques were also effective in terms of surgical radicality and local recurrence (Table 2) but VATS approach allowed a significantly lower chest tube length (11.89 ± 9.55 vs. 25.82 ± 24.37 days, *p*: 0.003) and post-operative stay (15.63 ± 11.69 vs. 25.53 ± 23.33, *p*: 0.018). The 30-day mortality for complications related to surgery (pneumonia or mediastinitis) was higher in open group (3 patients (15.7%) vs. 0, *p*: 0.002). The recorded level of pain on I post-operative day in Uniportal-VATS group was 1.89 ± 1.60 vs. 4.68 ± 2.91 in open group, *p*:  << 0.001.

The median FUP period was 35 months in the Uniportal-VATS series, while 52 months in the open one (median FUP of the whole series: 42 months). Twenty-one (28%) patients were lost at FUP, 8 (42%) from open group.

**Figure 2:**
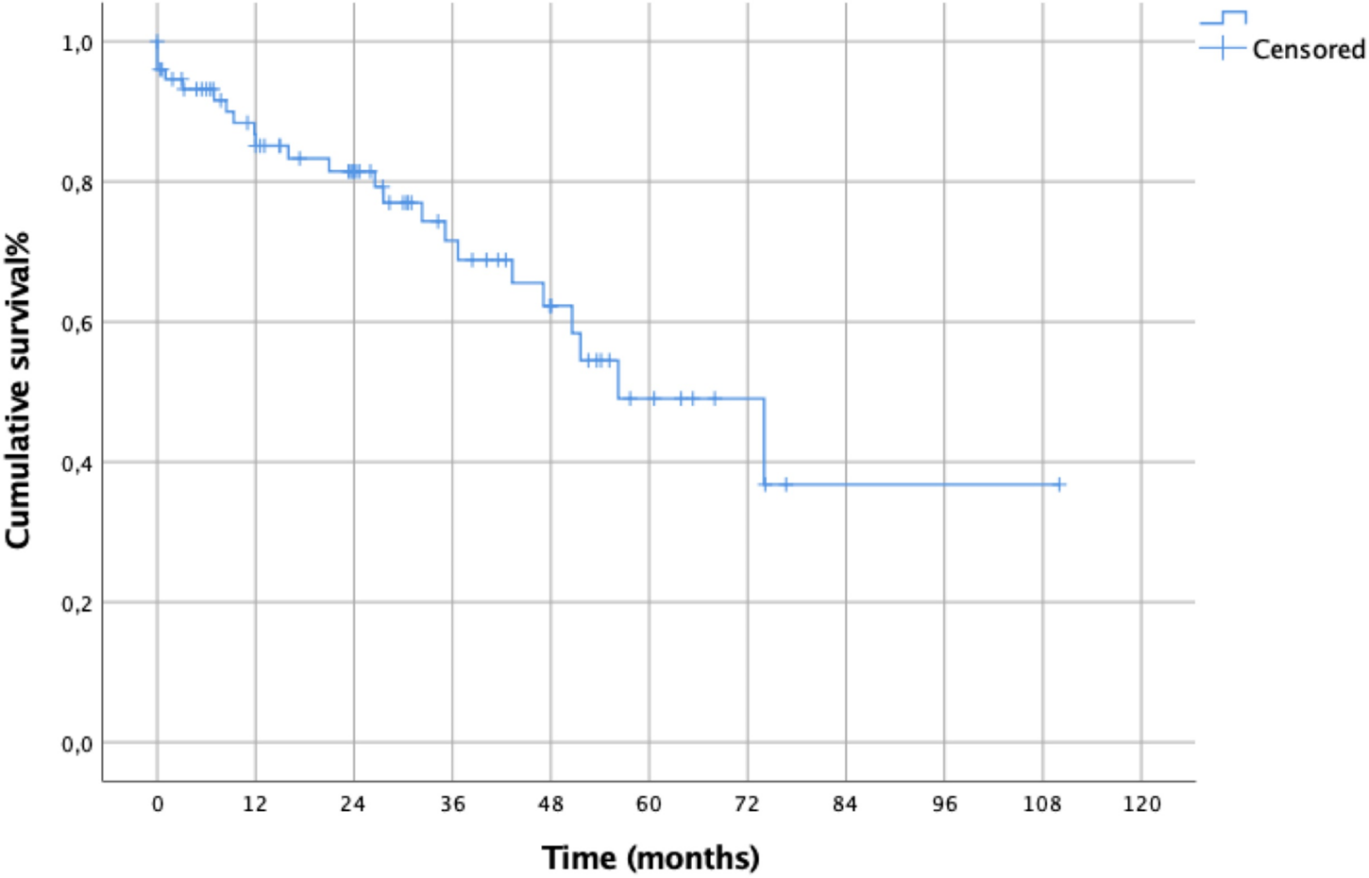
Overall survival of the whole population.

The 2-, 5- and 8-year survival of the whole series was 72%, 50% and 33%, respectively, Figure 2.

**Figure 3:**
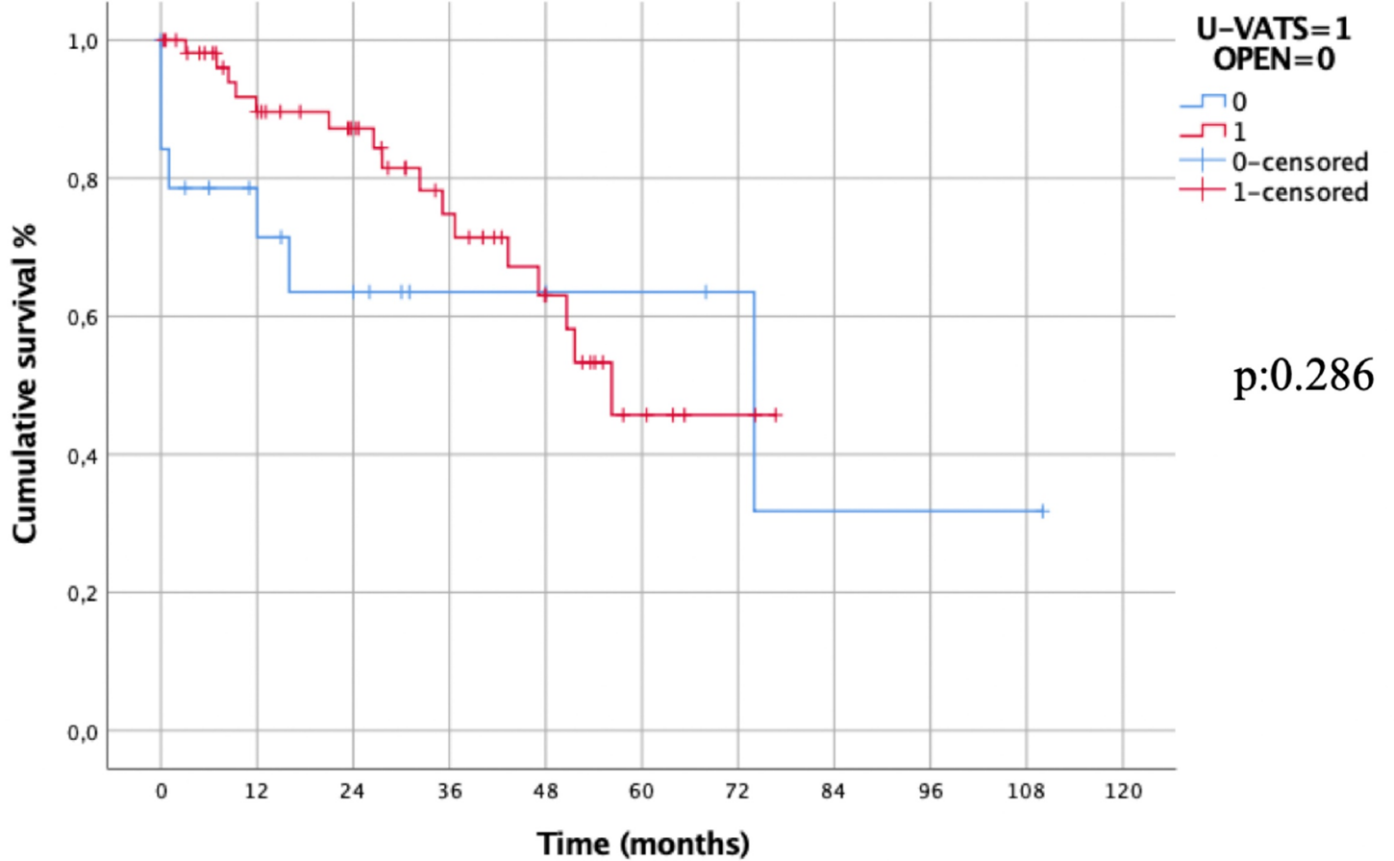
Overall survival in uniportal-VATS vs. open groups.

Combined 2- and 5-year OS in Uniportal- VATS group was 76% and 47% vs. 62% and 62% in open group, respectively (Log-rank test, *p*: 0.286, Figure 3). The results of Kaplan-Meier survival estimator model can be explained by the high number of events (deaths) recorded in open group during 60-days after surgery (4 events out of 7), while only 2 deaths for disease occurred during FUP period (with 8 patients lost). Therefore, the survival curves were also compared by Breslow-Wilcoxon test for having a more reliable analysis that took into account the events of the first period (*p*: 0.036).

No difference in OS was also recorded evaluating the survival per surgical approach in pathological stage I (*p*: 0.424) and II (*p*: 0.329), respectively. On the contrary, in stage III, the 5-year OS in Uniportal-VATS group was statistically superior than in open group (58% vs. 29%, *p*: 0.040). This was related to the fact that 6 out of 7 deaths in open group occurred among stage III patients, in particular the 4 patients died during first 60-days after surgery for complications.

**Figure 4:**
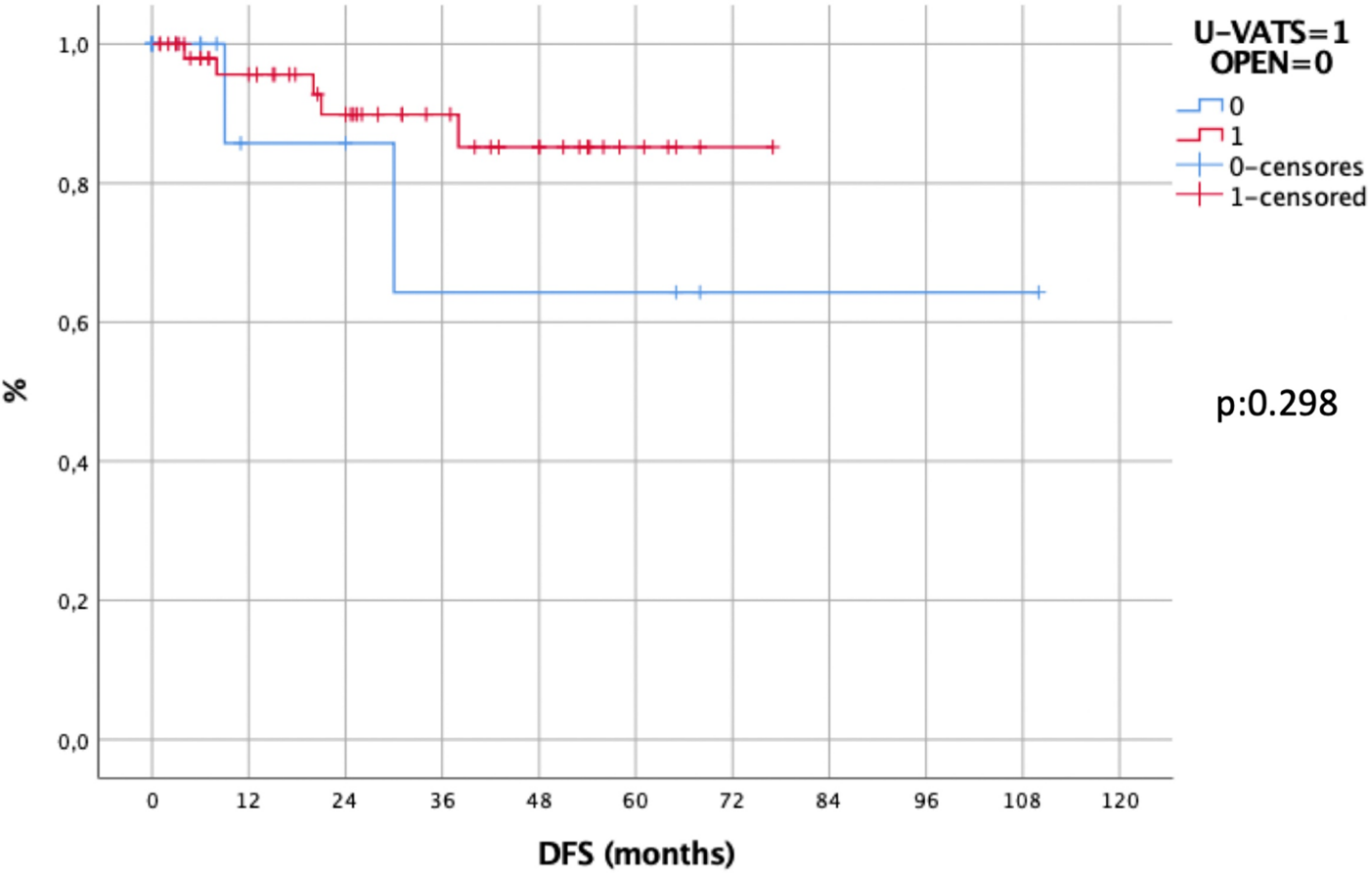
Disease-free survival in uniportal-VATS vs. open group.

No difference was recorded in DFS between the two approaches in general (5 year-DFS in Uniportal-VATS: 86% vs. 72%, *p*: 0.298, Figure 4) and per pathological stage II (*p*: 0.633) and III (*p*: 0.512), while in stage I the 5-year DFS in Uniportal-VATS was 100% vs. 60% in open, *p*: 0.019.

**Figure 5:**
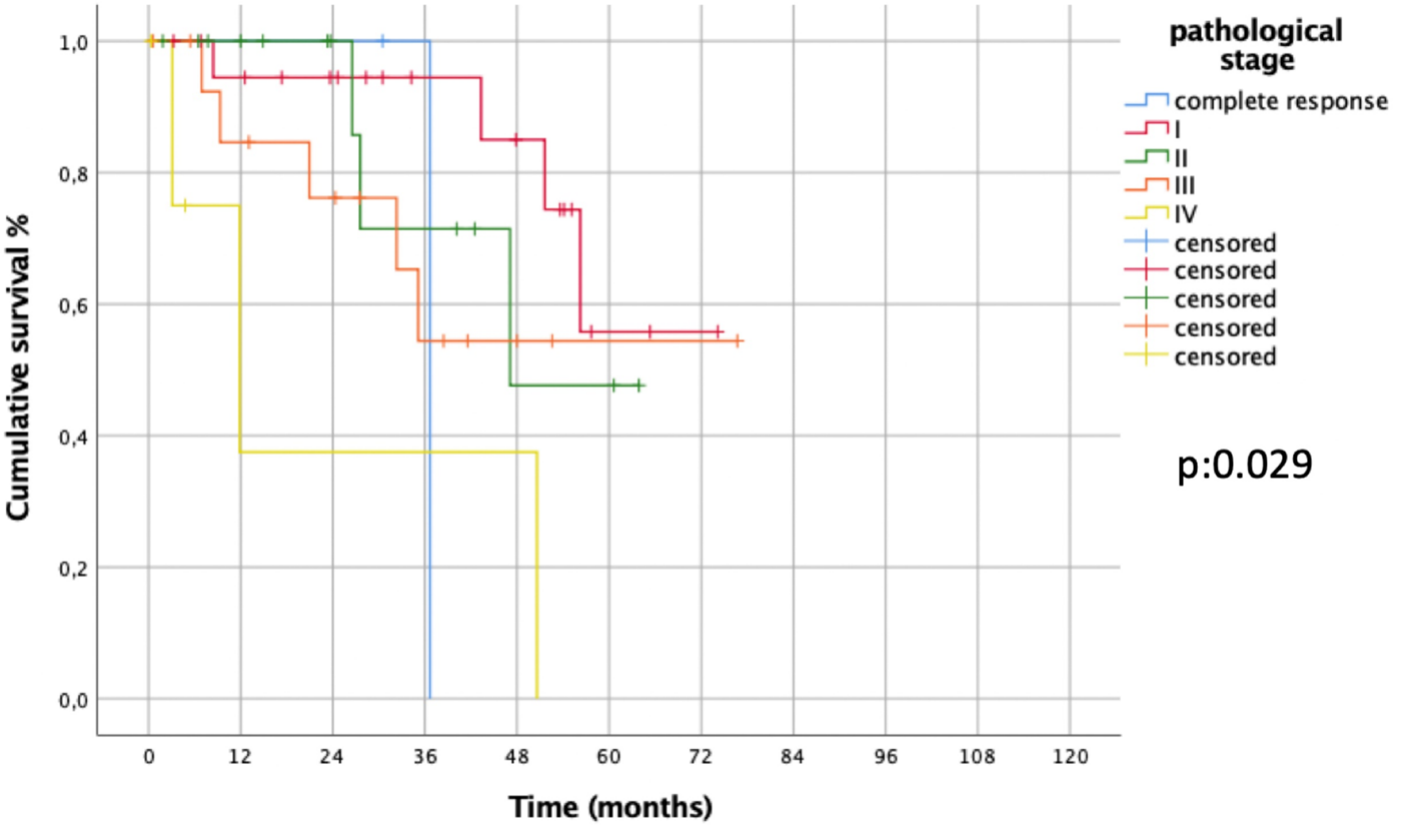
Overall survival in uniportal-VATS group per pathological stage.

Both in Uniportal-VATS group (*p*: 0.029, Figure 5) and open group (*p*: 0.006) the pathological stage significantly affected OS.

At multivariate Cox regression analysis, to assess factors independently affecting survival in the whole series, only pathological stage (stage I vs. other stages) confirmed its role (HR [95% CI]: 0.127 [0.022–0.723], *p*: 0.02), not the surgical approach (Uniportal-VATS vs. open: HR [95% CI]: 0.588 [0.226–1.529], *p*: 0.276).

## 4. Discussion

Till now, only small retrospective studies, mainly technical, have been published about Uniportal-VATS esophagectomy.

Therefore, it is of crucial importance having evidences about safety, surgical and oncological effectiveness of Uniportal-VATS approach compared to standard open technique or other MIEs.

After our preliminary series of 12 procedures (all McKeown) reported in 2018 (9), other authors confirmed the feasibility and efficacy of Uniportal-VATS approach for esophagectomy (3, 10).

The main used criteria for evaluating surgical and short-term outcomes of esophageal surgery are: duration of surgery, R0-resection, number of thoracic nodes removed and rate of anastomotic leak (3).

Batirel (3), in his preliminary series on 18 Uniportal-VATS esophagectomy (16 Ivor-Lewis and 2 McKeown), reported a mean number 23 ± 8 lymph nodes, with a mean VATS time of 82 ± 22 min. Three patients developed a leak (2 in the thorax and 1 in the neck). Similar results were reported on an updated series of 40 patients by the same group (VATS time: 90–100 min, lymph node yield: 20–25) (3). To date, the largest published series (10) on Uniportal-VATS (prone position) McKeown esophagectomy involved 44 cases, with a reported mean thoracic time of 163 ± 16 min and 24 (range: 14–36) nodes resected. All patients had a R0-resection; the mean hospital stay was 11.8 days (range:7–22), with 2 major complications descried and mortality null at 2-month FUP.

The only report comparing Uniportal-VATS esophagectomy short-term outcomes with a propensity-matched control group (multiportal MIE) was published by Lee (11) in 2017. Forty-eight patients undergone Uniportal-VATS (22 McKeown) for esophageal cancer were compared with 48 multiportal MIE patients. The authors concluded that both techniques were comparable in terms of duration of surgery, bleeding, total thoracic nodes retrieved and surgical complications (as anastomotic leak).

Only the pain-score one week after surgery was significantly lower in the Uniportal-VATS group (*p* < 0.05).

Our retrospective series of 56 McKeown esophagectomies is the largest reported with Uniportal-VATS approach and the first with a control group (open), from a single center prospectively recorded data. Moreover, in our study all patients underwent the same esophageal reconstruction (McKeown), excluding all Ivor-Lewis procedures (the other available comparison studies (11, 12) evaluated together McKeown and Ivor-Lewis esophagectomies) in order to reduce any bias related to different esophageal reconstruction (as anastomotic leaks) in comparing open and Uniportal-VATS approaches.

Furthermore, while all the previous papers dealt with only short-term outcomes, we also compared long-term oncological outcomes, with a median FUP period of 42 months.

According to our findings, Uniportal-VATS approach seemed comparable to standard open approach for McKeown esophagectomy in terms of thoracic surgical time (102.34 ± 15.21 vs. 115.56 ± 23.12 min, *p*: 0.646), nodes retrieved (13.40 ± 8.12 vs. 15.00 ± 6.86, *p*: 0.275), R + status (1 (1.8%) vs. 0, *p*: 0.655) and surgical complications, like anastomotic leak (4 (7.1%) vs. 4 (21.1%), *p*: 0.095), Table 2.

A superiority of Uniportal-VATS approach was recorded for a significantly lower re-operation rate (*p*: 0.004), chest drain duration (*p*: 0.003), in-hospital stay (*p*: 0.018), pain on I post-operative day (*p* << 0.001) and 30-day mortality (*p*: 0.002). Fifty percent of re-operations in open group was due to persistent chylothorax (in cases where surgical duct was not closed at time of esophagectomy), that was surgically treated after failure of conservative treatment (by exclusive parenteral nutrition for at least 7–10 days). Two cases of chylothorax were solved by conservative treatment only. Chylothorax, together with anastomotic leak (where one chest drain was kept in place precautionary till leak resolution) and the higher average of pleural effusion after thoracotomy explained the longer chest drain duration in open group.

In our Uniportal-VATS series, 58.9% of patients underwent neoadjuvant therapy and 57.1% concomitant radiotherapy, therefore this aspect did not discourage indication to minimally-invasive approach or cause conversion to open surgery.

Furthermore, in the hands of experienced Uniportal-VATS surgeons and high-volume centers in esophageal surgery, as in our series, Uniportal-VATS esophagectomy seems to have a quite short learning curve, with only 34 cases necessary to reach CLC and 40 cases for mastery.

Oncological outcomes, as 5-year OS (Log-rank test, *p*: 0.286; Breslow- Wilcoxon test, *p*: 0.036.) and DFS (*p*: 0.298) of patients undergone Uniportal-VATS esophagectomy were not inferior to those of standard treatment (open surgery), and the only factor independently affecting survival in our series was pathological stage (*p*: 0.02) not surgical access (*p*: 0.276).

Our results were in line with those reported by the TIME Trial (12), the only prospective randomized study comparing 56 open esophagectomies (McKeown and Ivor-Lewis) with 59 multiportal MIE, in terms of surgical and long-term oncological outcomes (12, 13). Indeed, Uniportal-VATS, as MIE in Time Trial, was superior to open surgery for in-hospital stay and post-operative pain, but comparable with open surgery for complications, nodal yeld and radicality, with similar long-term OS and DFS. This suggests that Uniportal-VATS approach allows comparable esophageal dissection as MIE and open surgery, without compromising long-term oncological outcomes, even after neoadjuvant chemoradiation, but with better post-operative recovery than thoracotomy.

From a purely technical point of view, we agree with Wang and colleagues (10) in performing the 4 cm Uniportal-VATS incision in the V intercostal space but more posteriorly than in lung surgery (14), between posterior and middle axillary line. But we believe that it is not necessary to put the patient in prone position (10) for easily and safely dissecting the posterior mediastinum. As Batirel (3), we strongly emphasize the importance of using surgical table as an instrument in this technique, for improving mediastinum exposure. In fact, tilting the patient on his ventral side of 30–45°, with 30–45° of anti-Trendelenburg position, we have no difficult at all in dissecting the esophagus, performing radical lymphadenectomy and managing several instruments through the same incision, without fencing. In our experience, we always used the V intercostal space, so we cannot support with our data the improvement reported by Batirel (3) by performing the incision in VI intercostal space.

The present study has several limitations. As single center, retrospective, non-randomized study, it is affected by several selection biases: the sample size is not large, and the control group is small (although both groups were statistically comparable for main clinic-pathological variables, as in Table 1), enrollment of patients in the 2 groups is time-depending (due to change in surgical approach –open vs. Uniportal-VATS- occurred at our center in 2016), and some survival data are lacking with 28% of patients lost at FUP (42% of which from open group).

However, to the best of our knowledge, this report is the first comparing surgical and oncological outcomes of only Uniportal-VATS and open McKeown esophagectomy (without involving other esophageal reconstruction techniques as Ivor-Lewis), with the largest Uniportal-VATS series reported in literature and longest oncological FUP (Median FUP: 42 months vs. 22 months of TIME Trial (13).

According to our results, Uniportal-VATS seems to be a safe, feasible and effective technique for performing McKeown esophagectomy, with equivalent surgical and long-term oncological results as the standard thoracotomy, but with a faster and unharmed recovery and a quite short learning curve.

Further prospective randomized trials with open and other minimally-invasive approaches are claimed to confirm the effectiveness of Uniportal-VATS in esophageal surgery.

## Data Availability

The raw data supporting the conclusions of this article will be made available by the authors, without undue reservation.
